# Robust reconstitution of active cell-cycle control complexes from co-expressed proteins in bacteria

**DOI:** 10.1186/1746-4811-8-23

**Published:** 2012-06-28

**Authors:** Hirofumi Harashima, Arp Schnittger

**Affiliations:** 1 Department of Molecular Mechanisms of Phenotypic Plasticity, Institut de Biologie Moléculaire des Plantes du CNRS, IBMP-CNRS - UPR2357, Université de Strasbourg, 12, rue du Général Zimmer, F-67084, Strasbourg Cedex, France; 2 Institut trinational pour la recherche sur les plantes, Institut de Biologie Moléculaire des Plantes du CNRS, IBMP-CNRS - UPR2357, Université de Strasbourg, 12, rue du Général Zimmer, F-67084, Strasbourg Cedex, France

## Abstract

**Background:**

Cell proliferation is an important determinant of plant growth and development. In addition, modulation of cell-division rate is an important mechanism of plant plasticity and is key in adapting of plants to environmental conditions. One of the greatest challenges in understanding the cell cycle of flowering plants is the large families of CDKs and cyclins that have the potential to form many different complexes. However, it is largely unclear which complexes are active. In addition, there are many CDK- and cyclin-related proteins whose biological role is still unclear, i.e. whether they have indeed enzymatic activity. Thus, a biochemical characterization of these proteins is of key importance for the understanding of their function.

**Results:**

Here we present a straightforward system to systematically express and purify active CDK-cyclin complexes from *E. coli* extracts. Our method relies on the concomitant production of a CDK activating kinase, which catalyzes the T-loop phosphorylation necessary for kinase activity. Taking the examples of the G1-phase cyclin CYCLIN D3;1 (CYCD3;1), the mitotic cyclin CYCLIN B1;2 (CYCB1;2) and the atypical meiotic cyclin SOLO DANCERS (SDS) in conjunction with A-, B1- and B2-type CDKs, we show that different CDKs can interact with various cyclins *in vitro* but only a few specific complexes have high levels of kinase activity.

**Conclusions:**

Our work shows that both the cyclin as well as the CDK partner contribute to substrate specificity in plants. These findings refine the interaction networks in cell-cycle control and pinpoint to particular complexes for modulating cell proliferation activity in breeding.

## Background

Understanding and modulating plant growth is of global significance due to the still rapidly increasing demand of food supply for the growing human population. In addition, there is a rising interest in plants as a source of renewable energy. An important parameter of plant body mass is the number of cells formed and thus, understanding plant cell proliferation is important beyond insights into the basic mechanism of cell division [1].

A substantial body of work has revealed a parts list of the core cell-cycle control in plants and general mechanisms of how these components are wired in regulatory networks are emerging [2-5]. In principle, Arabidopsis and other flowering plants contain, by and large, the same regulatory classes as found in animals and yeast, e.g. homologs of cyclin-dependent kinases (CDKs), cyclins, CDK inhibitors, the anaphase promoting complex/cyclosome (APC/C), E2F, Retinoblastoma, etc. Together with many functional studies on this gave rise to the notion that, similar to animals and yeast, specific CDK-cyclin complexes guide cells through the cell cycle, i.e. regulate progression from G1 (gap phase 1) into S phase (DNA replication phase) and from G2 (gap phase 2) into and through mitosis, where the sister chromatids are separated and distributed to the daughter cells.

One obvious difference between the plant cell cycle as opposed to the animal and yeast cell cycles, however, is the large number of regulatory proteins in different families, especially in the cyclin group. For instance, so far 10 A-type, 11 B-type and 10 D-type cyclins have been identified in the Arabidopsis genome [6,7]. In addition, there are a large number of CDK- and cyclin-like proteins, for example the putative cyclin SOLO DANCERS (SDS) that is more related to both A- and B-type cyclins than to D-type cyclins but has a very large N-terminal domain and only a degenerated destruction box (D-box), which typically mediates the proteolytic control of A- and B-type cyclins [8].

In addition to these quantitative differences, there are also in plants no obvious homologs for some of the key regulators of the animal cell cycle, foremost homologs of the phosphatase Cdc25 [9-11]. In turn, plants contain regulators that have no clear counterparts in animals or yeast, notably B-type CDKs. In Arabidopsis, the group of B-type CDKs contains four members divided in two subgroups, CDKB1 (CDKB1;1 and CDKB1;2) and CDKB2 (CDKB2;1 and CDKB2;2). In contrast to CDKA;1, which was found to rescue yeast cdc2 and cdc28 mutants [12-14], and which has a PSTAIRE cyclin binding signature as human Cdk1, Cdk2, and Cdk3, B-type CDKs are characterized by the plant-specific PPTALRE (in CDKB1) and PS/PTTLRE (in CDKB2) cyclin-binding motifs and at least CDKB1;1 (for CDKB2 there are no data available) did not rescue yeast cdc2 mutants [13,14]. Based on mutant phenotypes and expression of dominant negative versions, it has been suggested that CDKB1s especially control entry and progression through mitosis [15,16]. However, recent results indicated a function for the control into S-phase as well [4]. Consistent with the fact that CDKB1;1 and CDKB1;2 have an almost identical amino acid sequence, it was found that the two B1-type CDKs act largely redundantly and mutant phenotypes could only be found in the double mutant *cdkb1;1; cdkb1;2*[16].

Based on their expression peak during G2-M, the major time of action of CDKB2 kinases is probably in mitosis although a function outside of mitosis can currently not be ruled out [17]. Similar to the *CDKB1* group, *CDKB2s* are highly related and only the concomitant knock-down by artificial microRNAs resulted in plants with severe defects in the their shoot apical meristem function [18].

The view of specific CDK-cyclin complexes in plant cell-cycle regulation has recently been questioned by proteome-wide interaction assays revealing that most CDKs can bind to most cyclins resulting in dozens of different CDK-cyclin complexes [19]. This, together with the large classes of CDK-like and cyclin-like regulators, most of which remain poorly characterized, is a great challenge in the functional characterization of cell-cycle control in plants.

Here we present a rapid and robust biochemical assay system to test for enzymatic activity of specific CDK-cyclin complexes based on the comparison of different expression and purification systems. Our results show that only a fraction of all possible complexes have biological activity *in vitro* reducing the complexity of interaction data set,which will the identification of the specific CDK-cyclin combinations that are functional *in vivo*. In addition, our data show that substrate specificity is controlled by the specific composition of the CDK-cyclin complexes and that hence both partners contribute to target recognition in contrast to the typical situation in animals and yeast where the cyclin partner is thought to be the main target-determining factor.

## Results and discussion

### A duet vector system for efficient generation of active CDK-cyclin complexes in *E. Coli*

CDKs belong to the superfamily of protein kinases (PKs) that are characterized by a conserved protein fold, known as the PK fold [20]. Of key importance for kinase activity is the proper orientation of the T-loop in the C-terminal lobe. The T-loop gates access to the ATP and/or substrate binding pockets and participates in substrate recognition and orientation during the actual kinase reaction [21]. In the case of CDKs, crystallographic analyses of human Cdk2 with cyclin A have shown that both cyclin binding and phosphorylation of the T-loop are required for effective interaction of substrates with the kinase and hence for high levels of kinase activity [22]. This mechanism of T-loop action is also mandatory for activation of CDKA;1 in Arabidopsis [23,24].

Thus, the major challenge to assess CDK activity is its complex nature, i.e. the CDK kinase subunit must form a complex with a cyclin partner and the CDK must be activated by phosphorylation in the T-loop. T-loop phosphorylation is executed by a conserved class of kinases, so called CDK activating kinases (CAKs) that are found in all eukaryotes [25,26]. To circumvent the requirement of adding CAK activity in the expression system, insect cells have been successfully used to express and purify active CDK-cyclin complexes, including plant CDK-cyclin combinations [27].

However, insect cells also contain their endogenous CDKs, cyclins, and CDK inhibitors that may interfere with the kinase assays performed. This point may become crucial when assessing the many different CDK-cyclin combinations present in Arabidopsis and other plants of which some are expected to have perhaps only low levels of activity. Moreover, the insect cell system requires additional equipment and solutions that are cost intensive.

**Figure 1:**
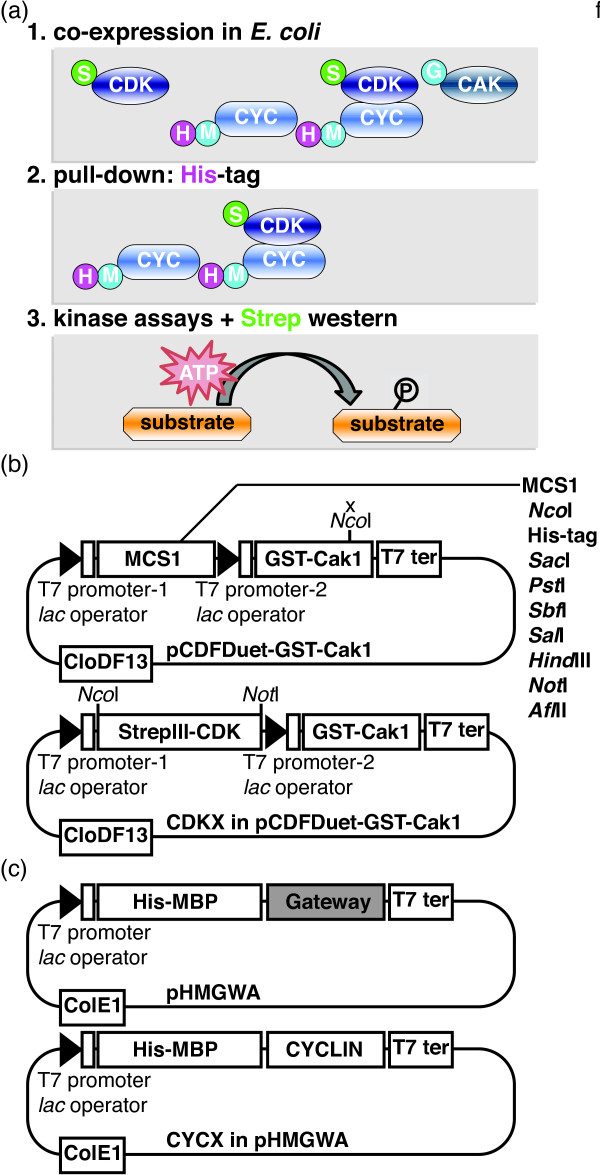
**Scheme of the here-developed kinase assay procedure based on a Duet-vector system.** (**a**) (**1**) CDK, e.g. CDKA;1, and cyclin, e.g. CYCD3;1, and CAK, e.g. Cak1 or CDKF;1, are co-expressed in *E. coli* and can be maintained due to the different replicons and the different resistances they confer, see also (**b**) and (**c**). (**2**) CDK-cyclin complexes, e.g. CDKA;1-CYCD3;1, are pulled-down through the His-tag on the cyclin partner. (**3**) Finally, kinase reactions are performed with the isolated complexes and complexes are in parallel subjected to western blotting with a Strep-Tactin-HRP detection system to determine the level of the CDK partner. (**b**) Generic Duet Vector with *CloDF13 ori* containing *Cak1* or *CDKF;1* (above) and Duet vector with *CDK* (*CDKA;1*/*CDKB1;1*/*CDKB2;2*) (below). (**c**) Generic Gateway-compatible Expression Vector with *ColE1 ori* (above) and vector with cyclin (*CYCD3;1*/*CYCB1;2*/*SDS*) (below).

Thus, we considered an *E. coli* expression system in which no endogenous CDKs or cyclins could interfere with the proteins of interest. However, as outlined above, such a system requires CAK activity to activate the CDK. We therefore used Cak1, the monomeric CAK from yeast *S. cerevisiae*. Unlike many CAKs, Cak1 requires neither binding of a cyclin partner nor an assembly factor for its activation nor other posttranslational regulatory steps [28]. This feature allowed us to reduce the number of vectors that need to be transformed: We generated a DUET vector to express both the kinase of interest, e.g. CDKA;1, fused to a StrepIII tag, and Cak1, which is monitored with a GST-tag (Figure 1a,b, Table 1). From a second vector with a compatible replicon, we expressed a HisMBP- or HisGST-tagged cyclin (Figure 1a,c, Table 1).

**Table 1:** Key vectors used in this study

**Vector properties**						
**Vector**	**Gene 1**	**Gene 2**	**Cloning sites (Gene1)**	**Cloning sites (Gene2)**	**Replicon**	**Antibiotic resistance**
pHGGWA	HisGST- CYCD3;1		Gateway		ColE1	Ampicillin
pHMGWA	HisMBP-SDS		Gateway		ColE1	Ampicillin
	HisMBP-CYCB1;2		Gateway		ColE1	Ampicillin
pHGGWA	HisGST- Wee1		Gateway		ColE1	Ampicillin
	HisGST- CDKF;1		Gateway		ColE1	Ampicillin
pCDFDuet-1	StrepIII- CDKA;1	GST-Cak1	*Nco*I - *Not*I	*Nde*I - *Xho*I	CloDF13	Spectinomycin
	StrepIII- CDKB1;1	GST-Cak1	*Nco*I - *Not*I	*Nde*I - *Xho*I	CloDF13	Spectinomycin
	StrepIII- CDKB2;2	GST-Cak1	*Nco*I - *Not*I (SLIC^†^)	*Nde*I - *Xho*I	CloDF13	Spectinomycin
		GST-Cak1		*Nde*I - *Xho*I	CloDF13	Spectinomycin
	StrepIII- CDKA;1		*Nco*I - *Not*I		CloDF13	Spectinomycin
		GST-CDKF;1		*Nde*I - *Xho*I	CloDF13	Spectinomycin
	StrepIII- CDKA;1	GST-CDKF;1	*NcoI - NotI*	*Nde*I - *Xho*I	CloDF13	Spectinomycin

^†^sequence and ligation-independent cloning.

**Figure 2:**
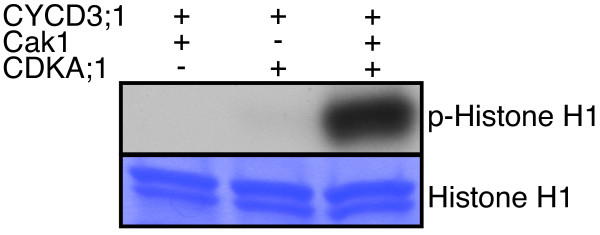
**Assessment of CDKA;1-CYCD3;1 activities from proteins purified from *E. coli *extracts.** Upper row, kinase assays against Histone H1 with plant proteins purified from bacteria. Complexes binding to a Ni-NTA column (His-tagged proteins) were purified from cells either expressing HisGST-tagged CYCD3;1 together with Cak1, or HisGST-CYCD3;1 together with StrepIII-tagged CDKA;1 or cells expressing all three components, i.e. CYCD3;1, CDKA;1 and Cak1. Only complexes from cells co-expressing all three proteins showed high levels of kinase activity demonstrating the requirement of T-loop phosphorylation of CDK-cyclin complexes. Note that the bacteria system has a very little background kinase activity. Lower row, the same SDS-PAGE gel stained with coomassie brilliant blue (CBB) reveals equal loading of Histone H1. Abbreviations: CBB Coomassie brilliant blue. p-Histone H1 for radio-labeled Histone H1 resulting from kinase assays with radio-labeled ATP.

To test this system, we generated a DUET vector with CDKA;1 and Cak1 along with HisGST-fused CYCD3;1 in the second vector. Next, Ni-NTA columns were used to purify recombinant protein complexes from *E. coli* extracts by means of the His-tag of the cyclins. Assessing the kinase activity of a CDKA;1-CYCD3;1 complex revealed very high levels of activity against the generic substrate Histone H1 from cells expressing all three components, i.e. CYCD3;1, CDKA;1 and Cak1, while CYCD3;1 complexes extracted from cells co-expressing Cak1 and CYCD3;1 or CDKA;1 were found to have almost no activity (Figure 2). Importantly, this *E. coli* system displayed very little background activity. This expression system was also efficient since we obtained approximately 100 μg CDKA;1 protein that was co-purified with CYCD3;1 from a 50-ml culture (see methods section, Additional file 1: Figure S1).

### Expression and purification of active CDKA;1-SDS complexes from *E. Coli*

**Figure 3:**
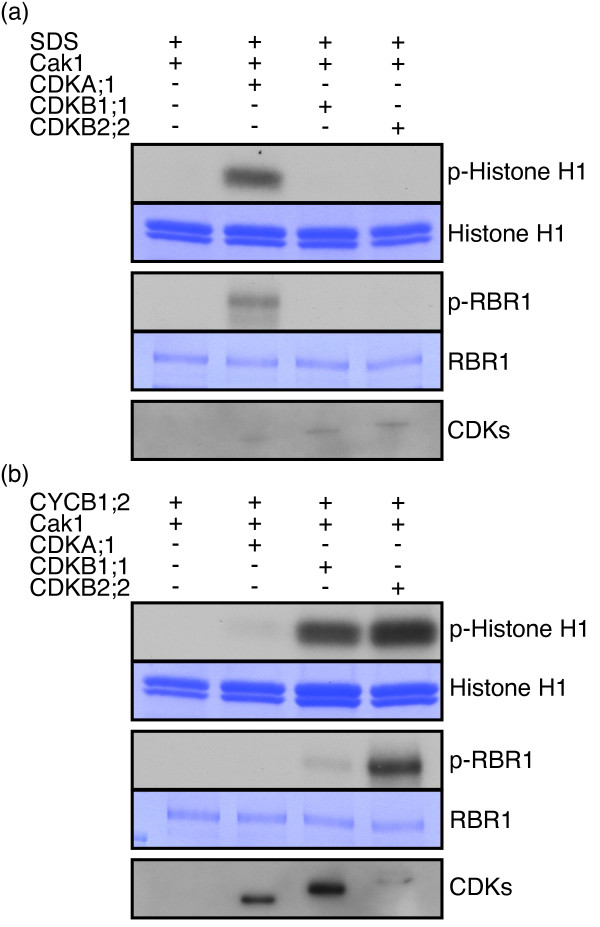
**Activity of SDS containing CDK-cyclin complexes.** For both panels, the first and third rows show kinase assays using bovine Histone H1 as a generic substrates as well as RBR1 from Arabidopsis that was expressed and purified from bacteria. Second and forth panels depict the results of CBB staining of the same SDS-PAGE gel indicating equal amount of substrates in each reaction. The fifth row (CDKs) shows western blot analyses of the respective pull-downs using Strep-Tactin HRP (either CDKA;1, CDKB1;1, or CDKB2;2 as indicted in the header of each panel) demonstrating that the respective CDKs bound to the particular cyclin that was co-expressed. (**a**) Complexes binding to a Ni-NTA column (His-tagged proteins) were purified from bacteria either expressing HisMBP-tagged putative cyclin SDS together with Cak1, or HisMBP-SDS together with Cak1 and StrepIII-tagged CDKA;1, or HisMBP-SDS together with Cak1 and StrepIII-tagged CDKB1;1, or HisMBP-SDS together with Cak1 and StrepIII-tagged CDKB2;2. Only CDKA;1-SDS but not CDKB1;1-SDS and CDKB2;2-SDS complexes isolated from *E. coli* extracts have high activity against both the generic substrate Histone H1 and Arabidopsis RBR1. (**b**) Complexes binding to a Ni-NTA column (His-tagged proteins) were purified from cells either expressing HisMBP-tagged cyclin CYCB1;2 together with Cak1, or HisMBP-CYCB1;2 together with Cak1 and StrepIII-tagged CDKA;1, or HisMBP-CYCB1;2 together with Cak1 and StrepIII-tagged CDKB1;1, or HisMBP-CYCB1;2 together with Cak1 and StrepIII-tagged CDKB2;2. CDKB1;1-CYCB1;2 and especially CDKB2;2-CYCB1;2 but only weakly CDKA;1-CYCB1;2 complexes isolated from *E. coli* extracts have activity against the two substrates Histone H1 and RBR1. Note that kinase assays between different panels, e.g. (**a**) and (**b**) cannot *a priori* be compared due to different levels of precipitated kinase complexes and the exposure time. Abbreviations: CBB Coomassie brilliant blue. p-Histone H1 for radio-labeled Histone H1 resulting from kinase assays with radio-labeled ATP.

To test whether our system can monitor CDK-cyclin complexes that have not previously been analyzed, in particular those involving CDK and or cyclin-like proteins, we decided to assess the activity of the putative cyclin SDS in combination with the three major core cell cycle CDKs, i.e. CDKA;1, CDKB1;1 and CDKB2;2. SDS is required for meiosis in Arabidopsis and has been found to interact with both CDKA;1 and CDKB1;1 in yeast two hybrid assays [8]. Consistent with previously reported results, we found that SDS interacted with both CDKA;1 and CDKB1;1 since we could precipitate StrepIII-CDKA;1 and StrepIII-CDKB1;1 with HisMBP-tagged SDS. In addition, we could precipitate StrepIII-CDKB2;2 with HisMBP-SDS. Next, we tested the biological activity of these three complexes in kinase assays against Histone H1. Notably, we found that SDS was only active in complexes with CDKA;1 (Figure 3a). Similar specificity was observed by using the Arabidopsis homolog of the animal retinoblastoma protein RBR1 as an alternative substrate (Figure 3a).

To verify whether active CDKB complexes could be generated in our system, we used CYCB1;2 as a cyclin partner. All three CDKs were found to be functional together with CYCB1;2 but the activity levels of CDKB2;2 were the highest, followed by CDKB1;1 and only then CDKA;1 using either Histone H1 or RBR1 as a substrate (Figure 3b). These findings are supported by genetic data showing that *cdkb1;1 cdkb1;2* double mutants do not have meiotic defects (for CDKB2 this analysis could not be done due to severe sporophytic defects in plants expressing amiRNA constructs against CDKB2) while already a reduction of CDKA;1 activity causes severe defects in meiosis [9,16,18,23].

### Substrate specificity is determined by the cyclin and the CDK partner in plants

CDK substrate specificity is thought to be largely determined by the cyclin partner as based on experiments in yeast and animals [29,30]. Interestingly, we observed a difference between CDKB1;1 and CDKB2;2 with respect to their relative activity against Histone H1 and RBR1 when pairing with the same cyclin partner, i.e. CYCB1;2. While both CDKB1;1 and CDKB2;2 in combination with CYCB1;2 showed high levels of activity against Histone H1, CDKB2;2-CYCB1;2 was much more effective to phosphorylate RBR1 than CDKB1;1-CYCB1;2 (Figure 3b). This differentiality demonstrated that the two complexes are not selectively activated by Cak1 but that the composition of the complexes, i.e. the specific CDK-cyclin combinations, is responsible for the substrate specificity. Moreover, since the cyclin partner was the same, these data indicate a key role of CDK unit for the activation of plant cell-cycle complexes.

**Figure 4:**
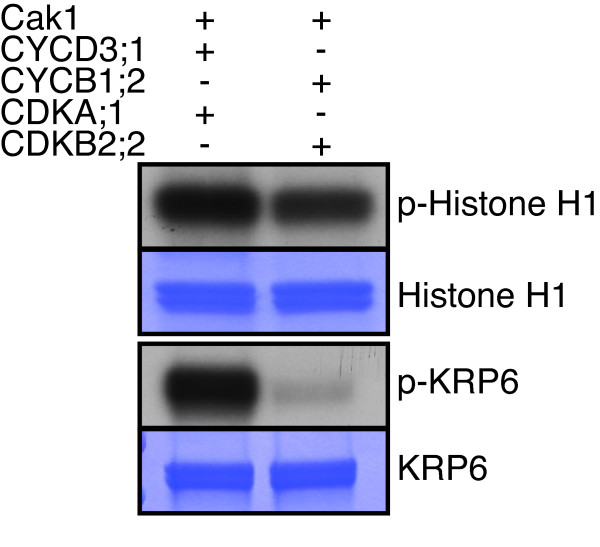
**Comparison of the kinase activity of CDKA;1-CYCD3;1 and CDKB2;2-CYCB1;2.** Upper row, the kinase activity of CDKA; 1-CYCD3;1 and CDKB2;2-CYCB1;2 were normalized against Histone H1. Complexes binding to a Ni-NTA column (His-tagged proteins) were purified from cells either expressing HisGST-CYCD3;1 together with StrepIII-CDKA;1 and Cak1, or HisMBP-CYCB1;2 together with StrepIII-CDKB2;2 and Cak1. Lower row, kinase assays against KRP6 with the kinases as indiated above. Proteins were subjected to SDS-PAGE after the kinase reaction and stained with coomassie brilliant blue (CBB) demonstrating equal protein loading of the substrate. Abbreviations: CBB Coomassie brilliant blue. p-Histone H1 and p-KRP6 for radio-labeled Histone H1 and KRP6, respectively, resulting from kinase assays with radio-labeled ATP.

To further address how the specificity of CDK-cyclin complexes is controlled in plants, we directly compared the two most active complexes that we identified in the course of our experiments, i.e. CDKA;1-CYCD3;1 and CDKB2;2-CYCB1;2 (Additional file 1: Figure S1). Based on their similar activity against Histone H1 both complexes were sufficiently activated by Cak1 (Figure 4). Next, we used another putative substrate, i.e. the CDK inhibitor KRP6 that contains one long (S/T-P-X-R/K) and three additional short (S/T-P) CDK-phosphorylation sites. Indeed, KRP6 could be phosphorylated by both CDK-CYC complexes *in vitro* indicating that it is a *bona fide* CDK substrate (Figure 4). Interestingly, KRP6 was equally strong phosphorylated as Histone H1 by CDKA;1-CYCD3;1. In contrast, CDKB2;2-CYCB1;2 had much higher activity against Histone H1 than against KRP6 in our *in vitro* system. Thus, the two different kinase complexes showed a specific activity profile. While one single CDK-cyclin combination has been found to be sufficient to drive the cell cycle in *Schizosaccharomyces pombe*, the specific activities of distinct CDK-cyclin complexes are thought to mediate cell-cycle progression in mammalian cells [31,32]. The here-obtained results indicate the cell cycle in plants likely follows the pattern found in multicellular eukaryotes.

### Expression and purification of active CDK-cyclin complexes from *E. Coli* using Arabidopsis CDKF;1 as CAK

**Figure 5:**
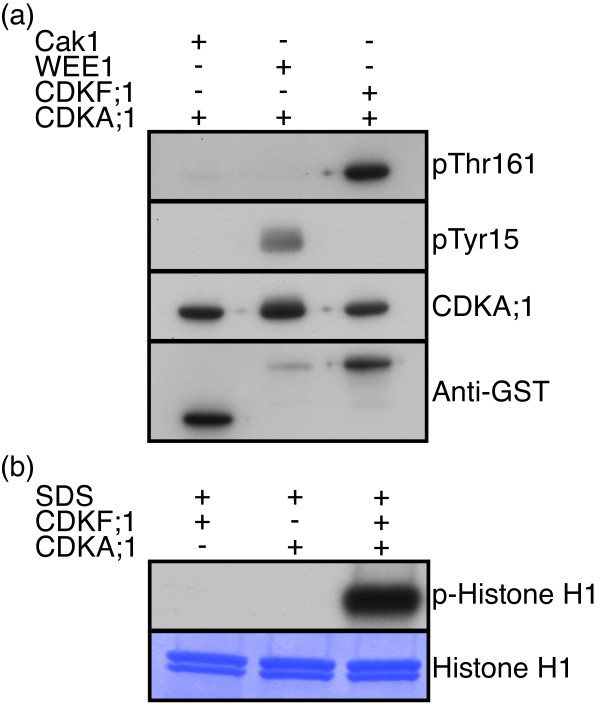
**Use of Arabidopsis CDKF;1 as a CAK. (a)** StrepIII-tagged CDKA;1 was co-expressed in bacteria with either GST-tagged Cak1 (from *S. cerevisiae*), or GST-tagged WEE1 (Arabidopsis), or GST-tagged CDKF;1 (Arabidopsis). The phosphorylation status of CDKA;1 was determined by western blots using antibodies against Phospho-Tyr (pTyr15), typically catalyzed by the kinase WEE1, and Phospho-Thr161 in the T-loop of CDKA;1 (pThr161), typically catalyzed by the activating kinase, either Cak1 or CDKF;1. Arabidopsis CDKF;1 can efficiently phosphorylate the T-loop of CDKA;1 at the residue T161 but not Y15 in the P-loop. In contrast, the kinase WEE1 can phosphorylate CDKA;1 at residue Y15 but not T161. **(b)** Complexes binding to a Ni-NTA column (His-tagged proteins) were purified from bacteria either expressing HisMBP-tagged SDS together with CDKF;1 as a CAK, or HisMBP-SDS together with StrepIII-tagged CDKA;1, or HisMBP-SDS together with CDKF;1 and StrepIII-tagged CDKA;1. High levels of kinase activity could be recovered from CDKA;1-SDS complexes that were activated by the endogenous Arabidopsis CAK, CDKF;1.

Although Cak1 could efficiently phosphorylate CDKs from Arabidopsis, a production system entirely relying on plant proteins would be favored, to resemble as much as possible the endogenous situation of plant cell-cycle control. Thus, we next replaced Cak1 with CDKF;1, a Cak1 homolog in Arabidopsis that functions as a monomer similar to Cak1, i.e. does not need a cyclin partner or other posttranslational modifications (Table 1) [33]. First, we tested whether CDKF;1 phosphorylated the T-loop of CDKA;1 in *E. coli*, using the kinase WEE1 that can phosphorylate CDKA;1 in the P-loop (Thr14 and/or Tyr15) as a negative control. Consistent with previous data [33-35], we found that WEE1 phosphorylated CDKA;1 at Tyr15 in the P-loop and that CDKF;1 phosphorylated CDKA;1 in the T-loop at the residue Thr161 as confirmed by western blot analysis using a phospho-specific antibodies (Figure 5a). Consistent with the result of CDKF;1 phosphorylation of CDKA;1, we retrieved high levels of CDKA;1-SDS activities in subsequent kinase reactions while CDKF;1 together with SDS showed no activity against Histone H1 (Figure 5b). Remarkably, we found that the ‘CAK’ activity of CDKF;1 against CDKA;1 was stronger than that of Cak1 under our experimental condition (note similar protein levels of Cak1 and CDKF;1 in Figure 5a). Possibly, this difference between Cak1 and CDKF;1 reflects some so far not recognized substrate specificity at the level of CAKs and reassures the use of the endogenous Arabidopsis CAK in kinase assays.

## Conclusions

Here we have developed an *in vitro* plant kinase assay system that uses only endogenous plant cell-cycle regulators. Due to its importance, many different methods have been developed to study protein phosphorylation [20,36]. Our system complements existing methods to monitor cell-cycle kinase activity, e.g. purification of general CDK activity exploiting CKS (Suc1 homolog)-coated beads or the precipitation of specific kinases, e.g. by means of specific tags followed by kinase assays. Each of these systems has particular strengths but also weaknesses. CKS-associated kinase assays are very suitable in mutant backgrounds when the expression of a tagged kinase version is not possible [4,37]. A drawback the CKS-associated kinase assays is that it is not clear whether and if so to what degree the beads discriminate against specific CDK-cyclin complexes or activation states. In contrast, precipitation of tagged kinases from plant extracts allows approximating the *in planta* activity of specific CDKs and/or cyclins. However, due to the large number of CDKs and in particular cyclins it is not easy to judge which CDK-cyclin complex(es) was isolated [38]. The here-presented system permits the analysis of definite CDK-cyclin complexes and a first and important result derived from the usage of this system is that only distinct CDK-cyclin complexes have high activity levels, at least *in vitro*. While binding of the cyclin and CDK partner is of course a prerequisite for activity, not all formed complexes, e.g. SDS with CDKB1;1 or CYCB1;2 with CDKA;1, appear to have (high) levels of activity and thus biological importance. Identifying active CDK-cyclin complexes reduces the number of relevant cell-cycle control complex tremendously. Our system will therefore be an important base to further explore the functions of the many CDK-cyclin regulatory pairs that possibly exist in Arabidopsis and other flowering plants [39]. Moreover, the assay will be important to explore the substrate space of plant CDKs, e.g. by determining the specific phosphorylation signatures – if they exist – and complement current attempts to identify kinase targets [38]. Finally, the system will be helpful in understanding kinetic and structural properties of functional CDK-cyclin complexes.

## Methods

All here generated material is freely available from the authors upon request including the vectors *pHGGWA-CYCD3;1*, *pHMGWA-SDS*, *pHMGWA-CYCB1;2*, *pCDFDuet-StrepIII-CDKA;1-GST-Cak1*, *pCDFDuet-StrepIII-CDKB1;1-GST-Cak1*, *pCDFDuet-StrepIII-CDKB2;2-GST-Cak1*, and *pCDFDuet-StrepIII-CDKA;1-GST-CDKF;1* (Table 1).

### Constructs

All manipulations were performed according standard molecular biological procedures [40]. The sequences of all primers used in this study can be found in Additional file 2: Table S1.

### StrepIII-CDKA;1 - CDKB1;1 - CDKB2;2

To obtain Strep-tag III (StrepIII) sequence, oligo nucleotides HH309 and HH310, HH311 and HH312, respectively, were annealed in 1X NEBuffer 3 (NEB) and phosphorylated by T4 polynucleotide kinase in 1X T4 DNA ligase buffer (Thermo scientific) and ligated into *Xho*I - *Hind*III sites of *pBluescript SK +* (Stratagene). To conjugate *attB1* recombination site and TEV protease recognition sequence at 5′ and 3′, respectively, of StrepIII, PCR was performed with primers HH323 and HH324. TEV protease recognition sequence and attB2 recombination site were introduced at 5′ and 3′, respectively, of *CDKA;1* by PCR with primers HH266 and HH268. The resulting PCR products were fused by PCR with primers HH323 and HH95, then recombined into *pDONR223* by using LR Clonase II (Invitrogen) to obtain *pDNOR223-StrepIII-CDKA;1*. The same strategies were taken to get *pDNOR223-StrepIII-CDKB1;1* and *pDNOR223-StrepIII-CDKB2;2*.

### Duet vectors

*GST-Cak1* was amplified by sequential PCR using *pGEX3C-cdk2-GST-Cak1*[41] as a template. First, to inactivate the *Nco*I recognition site in *Cak1*, *GST-Cak1* was amplified into two parts with primers HH393 and HH376 and primers HH383 and HH394 (Table 1). To fuse the two PCR products, PCR was performed with primers HH383 and HH376, and the products were digested with *Bgl*II and *Xho*I and ligated into *Bgl*II - *Xho*I sites of *pCDFDuet-1* (Novagen) to obtain *pCDFDuet-Cak1*. To conjugate GST-tag to CDKF;1, *GST* and *CDKF;1* cDNAs were amplified with primers HH667 and HH670, HH669 and HH668, respectively. The resulting PCR products were fused by PCR with primers HH667 and HH668. PCR products were digested with *Nde*I and *Xho*I and ligated into *Nde*I - *Xho*I sites of *pCDFDuet-1* to obtain *pCDFDuet-CDKF;1*. A StrepIII-tagged *CDKA;1*, *CDKB1;1* or *CDKB2;2* linked by a TEV protease recognition sequence were amplified by primers HH395 and HH396, HH395 and HH397 or HH398 and HH401, respectively. For *CDKA;1* and *CDKB1;1*, PCR products were digested with *Nco*I and *Not*I and ligated into *Nco*I - *Not*I sites of *pCDFDuet-Cak1* and *pCDFDuet-CDKF;1*; for *CDKB2;2*, PCR products were cloned into *Nco*I - *Not*I sites of *pCDFDuet-Cak1* and *pCDFDuet-CDKF;1* by SLIC [42].

### HisGST-CYCD3;1, HisMBP-SDS, CYCB1;2

*pDONR221-CYCD3;1* was kindly provided by Lieven De Veylder. *SDS* cDNA was kindly provided by Petra Bulankova and Karel Riha. To introduce *attB* recombination sites to *SDS*, PCR was performed with primers HH509 and HH510, then HH347 and HH348, sequentially. To introduce *attB* recombination sites to *CYCB1;2*, PCR was performed with primers HH339 and HH340, then HH347 and HH348, sequentially on the *CYCB1;2* cDNA [43,44] The resulting PCR products were recombined into *pDNOR223* and then recombined into a destination vector *pHGGWA* or *pHMGWA*[45] by using Gateway technology (Invitrogen) to fuse a HisGST- or HisMBP-tag, respectively.

### Wee1

*pDNOR201-Wee1* and *pDNOR201-CDKF;1* were kindly provided by Annika K. Weimer and Stefan Pusch, respectively. Recombination reaction were performed between the entry clone and a destination vector *pHGGWA* to fuse a HisGST-tag, resulting in *pHGGWA-Wee1* and *pHGGWA-CDKF;1*.

### RBR1

To express and purify GST-AtRBR1-His_6_, first *pGEX-4 T-1* (GE Healthcare) was amplified with primers HH294 and HH295 and self-ligated to introduce a hexa-histidine sequence (*pGEX-His*_*6*_). A full-length AtRBR1 was amplified with primers HH194 and HH293. PCR products were digested with *Bam*HI and *Xho*I, and ligated into *Bam*HI - *Xho*I sites of *pGEX-His*_*6*_ (*pGEX-AtRBR1-His*_*6*_).

### KRP6

*pDNOR201-KRP6* were kindly provided by Stefan Pusch. To express and purify HisGST-KRP6, a recombination reaction were performed between the entry clone and a destination vector *pHGGWA* to fuse a HisGST-tag, resulting in *pHGGWA-KRP6*.

### Phosphorylation of CDKA;1 in *E. Coli*

CDKA;1 proteins were produced in *E. coli* co-expressing either GST-fused Wee1, Cak1 or CDKF;1. The two proteins were co-expressed by transforming an *E*. *coli* SoluBL21 strain (AMS Biotechnology) with plasmids, *pCDFDuet-CDKA;1* and *pHGGWA-Wee1* or *pHGGWA-CDKF;1*; for co-expression of CDKA;1 and Cak1, *pCDFDuet-CDKA;1-Cak1* was used. Cells were grown in LB medium containing appropriate antibiotics to OD_600_ of 0.6 at 37°C. The production of proteins was induced by adding IPTG to 1 mM, cells were then cultured for 3 h at 37°C before being harvested by centrifugation. Cell pellets were boiled in 1X SDS-PAGE sample buffer (62.5 mM Tris-Cl, pH 6.8, 2%(w/v) SDS, 10%(v/v) glycerol, 1% ß-mercaptoethanol, 0.005% bromophenol blue) and the supernatant were conducted to the protein blotting. After separating the proteins on a 10% SDS-PAGE gel (SDS-PAGE running buffer (25 mM Tris, 192 mM glycine, 0.1%(w/v) SDS)), proteins were transferred onto a polyvinylidenfluorid membrane (Millipore) in a modified towbin buffer (SDS-PAGE running buffer containing 15%(v/v) methanol) with a wet blotting system (Bio-rad) [46]. To detect pTyr15 and pThr161, membrane was blocked with protein-free T20 (TBS) blocking buffer (Thermo scientific) and probed with a 1:5000 and 1:2000 dilution of phospho-cdc2 (Tyr15) (10A11) (Cell signaling) and phospho-cdc2 (Thr161) (Cell signaling), respectively as primary antibodies in protein-free T20 (TBS) blocking buffer and a 1:100000 dilution of horseradish peroxidase-conjugated anti-rabbit antibody (GE Healthcare) as a secondary antibody in 1%(w/v) non-fat dry milk in TBST. To detect StrepIII- and GST-tagged proteins, membrane was blocked with 5%(w/v) non-fat dry milk in TBST and probed with a 1:250000 and 1:20000 dilution of Strep-Tactin HRP and anti-GST HRP (GE Healthcare), respectively. In the case of StrepIII-tagged protein detection, biotin blocking buffer (IBA) was added at 1:1000 dilution prior to the Sterp-Tactin HRP incubation. Enhanced chemoluminescent detection was performed with HRP substrate (Millipore).

### Preparation of cyclin/CDK complexes in *E. Coli*

CDK-cyclin complexes were produced in *E. coli* co-expressing GST-fused *Saccharomyces cerevisiae* Cak1. All three proteins were co-expressed by transforming an *E*. *coli* SoluBL21 strain with the two plasmids, *pCDFDuet-1*, containing a *StrepIII-CDK* and *GST-Cak1*, and *pHGGWA* or *pHMGWA*, containing the respective cyclin. Cells were grown in 50 ml of LB medium containing 50 μg/ml ampicillin and 50 μg/ml spectinomycin at 37°C to OD_600_ of 0.6, and incubated for another 30 min at 18°C. The production of proteins was induced by adding IPTG to 0.3 mM, cells were then cultured overnight at 18°C before being harvested by centrifugation.

The cell pellet was re-suspended in 2.5 ml Ni-NTA binding buffer (50 mM NaH_2_PO_4_, 100 mM NaCl, 10%(v/v) glycerol, 25 mM imidazole, pH 8.0.) and lysed by sonication. After addition of Triton X-100 to 0.2%(w/v), the cell slurry was incubated with gentle agitation for 20 min at 4°C then centrifuged at 10,000 × g for 40 min at 4°C. The supernatant was passed through a 0.45 μm filter. The cleared lysates were applied onto a Econopack column (Bio-rad) packed with 300-μl Ni-NTA resin (Qiagen), which was washed sequentially with 3 ml Ni-NTA binding buffer, and eluted with 600 μl Ni-NTA elution buffer (50 mM NaH_2_PO_4_, 100 mM NaCl, 10%(v/v) glycerol, 225 mM imidazole, pH 8.0.). The buffer was exchanged to kinase buffer (50 mM Tris–HCl, pH 7.5, 10 mM MgCl_2_, 1 mM EGTA) containing protease inhibitors cocktail (Roche) and Phos-STOP (Roche) with a PD MiniTrap G-25 column (GE Healthcare). Purified kinases were frozen in liquid N2 and stored at −80°C until use.

### Preparation of the substrates

Histone H1 was purchased from Millipore. *E*. *coli* BL21-AI (Invitrogen) cells were transformed with the plasmid, *pGEX-AtRBR1-His*_*6*_ or *pHGGWA-KRP6* and grown until OD_600_ = 0.6 at 37°C. The culture was transferred to 18°C and grown for 30 min. The production of the fusion protein was induced by adding 0.3 mM IPTG and 0.2%(w/v) L-arabinose overnight at 18°C. Cells were harvested by centrifugation and re-suspended in phosphate-buffered saline (PBS) buffer (140 mM NaCl, 2.7 mM KCl, 10.1 mM Na_2_HPO_4_, 1.8 mM KH_2_PO_4_, pH 7.3), and lysed by sonication. After addition of Triton X-100 to 0.2%(w/v), the cell slurry was incubated at 4°C then clarified by centrifugation. The supernatant was passed through a column packed with Glutathione-agarose (Sigma), which was washed sequentially with PBS, and eluted with GST elution buffer (50 mM Tris–HCl, pH 8.0, 10 mM Glutathione). The eluate was sequentially purified with a column packed with Ni-NTA resin. GST-AtRBR1-His_6_ was eluted with Ni-NTA elution buffer and the buffer was exchanged to kinase buffer with a PD-10 column (GE Healthcare). Substrates were aliquoted and frozen in liquid N_2_ and stored at −80°C until use.

### Kinase assay

CDK-cyclin complexes were processed for kinase assays as described previously [27] with Histone H1, GST-AtRBR1-His_6_ or GST-KRP6 as a substrate. Loading of equal amounts of CDKA;1, CDKB1;1 and CDKB2;2 was assured by quantifying them by western blot with Strep-Tactin HRP.

## Authors’ contributions

Conceived and designed the experiments: HH and AS. Performed the experiments: HH. Analyzed the data: HH and AS. Wrote the paper: HH and AS. All authors read and approved the final manuscript.

## Competing interests

The authors declare no competing interest.

## Supplementary Material

Additional file 1 **Figure S1. **Semi quantitative estimation of CDKA;1-CYCD3;1 and CDKB2;2-CYCB1;2 protein amounts. Starting from a 50-ml *E. coli *culture, purified CDK-cyclin complexes were dissolved in a final volume of 1 ml kinase buffer. 3.75 μl of each sample was subjected to SDS-PAGE and the gel was stained with CBB. lane 1; 1 mg/ml BSA, lane 2; 0.75 mg/ml BSA, lane 3; 0.5 mg/ml BSA, lane 4; 0.25 mg/ml BSA, lane 5; 0.125 mg/ml BSA, lane 6; CDKA;1-CYCD3;1, lane 7; CDKB2;2-CYCB1;2. Green arrow head indicates cyclins, red arrow heads CDKs. Click here for file

Additional file 2 **Table S1. **Primer sequences. Click here for file
