# Supplementary figures and images for: Robust reconstitution of active cell-cycle control complexes from co-expressed proteins in bacteria
Source: Plant Methods. 2012 Jun 28;8:23. doi: 10.1186/1746-4811-8-23 (PMC3490756; doi:10.1186/1746-4811-8-23)

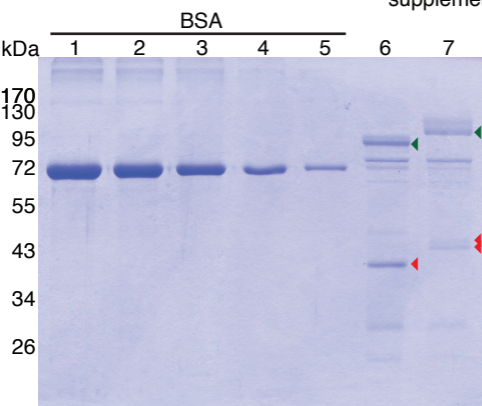

Supplement: Additional file 1 — Figure S1. Semi quantitative estimation of CDKA;1-CYCD3;1 and CDKB2;2-CYCB1;2 protein amounts. Starting from a 50-ml E. coli culture, purified CDK-cyclin complexes were dissolved in a final volume of 1 ml kinase buffer. 3.75 μl of each sample was subjected to SDS-PAGE and the gel was stained with CBB. lane 1; 1 mg/ml BSA, lane 2; 0.75 mg/ml BSA, lane 3; 0.5 mg/ml BSA, lane 4; 0.25 mg/ml BSA, lane 5; 0.125 mg/ml BSA, lane 6; CDKA;1-CYCD3;1, lane 7; CDKB2;2-CYCB1;2. Green arrow head indicates cyclins, red arrow heads CDKs. [file 1746-4811-8-23-S1.pdf]
