# Supplementary material for: Robust reconstitution of active cell-cycle control complexes from co-expressed proteins in bacteria
Source: Plant Methods. 2012 Jun 28;8:23. doi: 10.1186/1746-4811-8-23 (PMC3490756; doi:10.1186/1746-4811-8-23)
Supplement: Additional file 2 — Table S1. Primer sequences. [file 1746-4811-8-23-S2.pdf]

Supplementary Table 1. Vector properties

Vector	Gene 1	Gene 2	Cloning sites (Gene1)	Cloning sites (Gene2)	Replicon	Antibiotic resistance
pHMGWA	HisMBP-SDS	-	Gateway	-	ColE1	Ampicillin
	HisMBP-CYCB1;2	-	Gateway	-	ColE1	Ampicillin
pHGGWA	HisGST-CYCD3;1	-	Gateway	-	ColE1	Ampicillin
	HisGST-Wee1	-	Gateway	-	ColE1	Ampicillin
	HisGST-CDKF;1	-	Gateway	-	ColE1	Ampicillin
pCDFDuet-1	StrepIII-CDKA;1	GST-Cak1	*Nco*I - *Not*I	*Nde*I - *Xho*I	CloDF13	Spectinomycin
	StrepIII-CDKB1;1	GST-Cak1	*Nco*I - *Not*I	*Nde*I - *Xho*I	CloDF13	Spectinomycin
	StrepIII-CDKB2;2	GST-Cak1	*Nco*I - *Not*I (SLIC†)	*Nde*I - *Xho*I	CloDF13	Spectinomycin
	-	GST-Cak1	-	*Nde*I - *Xho*I	CloDF13	Spectinomycin
	StrepIII-CDKA;1	-	*Nco*I - *Not*I	-	CloDF13	Spectinomycin
	-	GST-CDKF;1	-	*Nde*I - *Xho*I	CloDF13	Spectinomycin
	StrepIII-CDKA;1	GST-CDKF;1	*Nco*I - *Not*I	*Nde*I - *Xho*I	CloDF13	Spectinomycin

†Sequence and ligation-independet cloning
